# Roles of Impurity Levels in 3d Transition Metal-Doped Two-Dimensional Ga_2_O_3_

**DOI:** 10.3390/ma17184582

**Published:** 2024-09-18

**Authors:** Hui Zeng, Chao Ma, Xiaowu Li, Xi Fu, Haixia Gao, Meng Wu

**Affiliations:** 1 College of Science, Hunan University of Science and Engineering, Yongzhou 425199, China; 2 College of Materials Science and Engineering, Hunan University, Changsha 410082, China; 3 Fujian Provincial Key Laboratory of Semiconductors and Applications, Collaborative Innovation Center for Optoelectronic Semiconductors and Efficient Devices, Department of Physics, Xiamen University, Xiamen 361005, China

**Keywords:** first principles, 2D Ga_2_O_3_, 3d TM, p-type conductivity, magnetic moment

## Abstract

Doping engineering is crucial for both fundamental science and emerging applications. While transition metal (TM) dopants exhibit considerable advantages in the tuning of magnetism and conductivity in bulk Ga_2_O_3_, investigations on TM-doped two-dimensional (2D) Ga_2_O_3_ are scarce, both theoretically and experimentally. In this study, the detailed variations in impurity levels within 3d TM-doped 2D Ga_2_O_3_ systems have been explored via first-principles calculations using the generalized gradient approximation (GGA) +U method. Our results show that the Co impurity tends to incorporate on the tetrahedral GaII site, while the other dopants favor square pyramidal GaI sites in 2D Ga_2_O_3_. Moreover, Sc^3+^, Ti^4+^, V^4+^, Cr^3+^, Mn^3+^, Fe^3+^, Co^3+^, Ni^3+^, Cu^2+^**,** and Zn^2+^ are the energetically favorable charge states. Importantly, a transition from n-type to p-type conductivity occurs at the threshold Cu element as determined by the defect formation energies and partial density of states (PDOS), which can be ascribed to the shift from electron doping to hole doping with respect to the increase in the atomic number in the 3d TM group. Moreover, the spin configurations in the presence of the square pyramidal and tetrahedral coordinated crystal field effects are investigated in detail, and a transition from high-spin to low-spin arrangement is observed. As the atomic number of the 3d TM dopant increases, the percentage contribution of O ions to the total magnetic moment significantly increases due to the electronegativity effect. Additionally, the formed 3d bands for most TM dopants are located near the Fermi level, which can be of significant benefit to the transformation of the absorbing region from ultraviolet to visible/infrared light. Our results provide theoretical guidance for designing 2D Ga_2_O_3_ towards optoelectronic and spintronic applications.

## 1. Introduction

2D nanomaterials are a novel family of materials that display remarkable qualities and present numerous prospects for use in optoelectronics and next-generation electronics such as graphene, h-BN, MoTe_2,_ etc. [1,2,3]. Recently, due to the fragile Ga-O bonds along the β-Ga_2_O_3_ [100] direction, two-dimensional (2D) Ga_2_O_3_ has gained considerable attention. It can be fabricated by applying mechanical exfoliation, molecular beam epitaxy, and chemical synthesis methods [4,5]. Due to the increased surface-to-volume ratio of 2D Ga_2_O_3_, its properties have been improved compared to its bulk counterpart, holding great potential for innovative nanoscale applications. For instance, a 2D Ga_2_O_3_-based solar-blind photodetector is boosted by a responsivity of about 1.8 × 10^5^ A/W, which is nearly three times higher than that of bulk β-Ga_2_O_3_ [6,7,8]. The breakdown voltage of the field-effect transistors increases from 113 V in bulk β-Ga_2_O_3_ to 344 V in 2D β-Ga_2_O_3_ [9,10]. However, similar to bulk β-Ga_2_O_3_, achieving p-type doping remains a challenge in 2D β-Ga_2_O_3_ because of the flat valence band maximum (VBM) composed mainly of O-2p orbitals, along with the intrinsic n-type defects (like oxygen vacancy (V_O_)) and unintentionally introduced dopants (such as C, Si, and Ir), which significantly limit its optoelectronic applications [11,12,13,14]. On the other hand, pristine β-Ga_2_O_3_ is characterized by a nonmagnetic ground state, which drastically hinders the prospects for spintronic devices. Therefore, exploring the abilities of p-type conductivity and magnetism in 2D β-Ga_2_O_3_ are essential to meeting the needs of emerging optoelectronic and spintronic applications.

Doping engineering is a powerful way to manipulate the conductivity ability and magnetic properties for wide-band gap semiconductors [15,16,17,18]. While studies on the p-type conductivity and magnetism of 2D Ga_2_O_3_ are scarce, both theoretically and experimentally, 3d TM elements have been identified as promising dopants, as suggested by the research on 3D Ga_2_O_3_. In the 3D Ga_2_O_3_ system, two acceptor impurity levels were created in Cu-doped β-Ga_2_O_3_, showing potential as a p-type semiconductor, as determined through first-principles calculations [19]. Additional analyses using electron paramagnetic resonance spectroscopies indicated that Cu^2+^ preferred to be positioned on the octahedral Ga site [20]. Zn-doped β-Ga_2_O_3_ owned shallow acceptor levels, demonstrating a p-type conductivity [21]. Wang et al. investigated the natures of impurity levels in (3d-5d) TM-doped α-Ga_2_O_3_ (the second stable Ga_2_O_3_ structure) and illustrated that the donor levels were variable with respect to the TM dopants for the electron-rich cases [22]. Gao et al. studied the 3d TM-doped β-Ga_2_O_3_ and indicated that the Ti dopant can be easily formed in the β-Ga_2_O_3_ system, followed by V, Cr, Sc, Fe, Mn, Co, Ni, Cu, and Zn elements, and all doping arrangements resulted in a redshift of β-Ga_2_O_3_ [23]. However, most of the studies lack detailed analyses of the roles of impurity levels within 3d TM-doped Ga_2_O_3_. Additionally, studies have not addressed the defect formation energies with different charge states and transition energy levels, which are vital to evaluate the p-type/n-type doping and the shallow/deep impurity ionization levels.

Moreover, 3d TM dopants are not only expected to achieve p-type conductivity, but they also provide the potential to introduce magnetism. Substituting some host atoms with TM elements in a nonmagnetic semiconductor like Ga_2_O_3_ can form a diluted magnetic semiconductor (DMS), which is extensively utilized in spintronic applications such as spin-light emitting diodes and spin-field effect transistors [24]. Therefore, identifying candidate DMS in the 2D Ga_2_O_3_ system is essential for broadening its spintronic applications. This study investigated the electronic and magnetic characteristics of V-, Cr-, and Mn-doped 2D Ga_2_O_3_ via first-principles calculations. These illustrated that the V, Cr, and Mn dopants introduced magnetic moments of 2, 3, and 4 μ_B_ into the 2D Ga_2_O_3_ systems, respectively [25]. For other 3d dopants, magnetic moments of 2.0 μ_B_ and 1.0 μ_B_ were detected in Cu- and Zn-doped 3D β-Ga_2_O_3_, respectively [19,26]. It was predicted that the Ni dopant preferentially incorporated on the octahedral Ga site in 3D β-Ga_2_O_3,_ accompanied by a 1.0 μ_B_ magnetic moment [27]. Ti-doped bulk β-Ga_2_O_3_ exhibited a magnetic moment of 0.546 μ_B_ and the induced Ti atom tended to replace the octahedrally coordinated Ga atom [28].

We note that the systematic studies regarding the role of 3d impurities in modifying the p-type conductivity and magnetism of 2D Ga_2_O_3_ material are absent. Especially considering the different local crystal structures between 2D and 3D Ga_2_O_3_, the results presented in 3D Ga_2_O_3_ are difficult to directly apply to 2D systems. In particular, each bulk β-Ga_2_O_3_ has octahedral GaI and tetrahedral coordinated GaII sites. By cleaving the Ga–O bonds to form 2D Ga_2_O_3_, GaI is restructured from six coordination to five coordination, featuring a square pyramidal structure. The variable crystal field can generate diverse electronic structures and doping effects, leading to different electric and magnetic properties for 2D Ga_2_O_3_ compared to its bulk counterpart. This motivates a comprehensive analysis of the effects of 3d orbitals on the magnetic properties and electronic structures of 2D Ga_2_O_3_.

In this study, we examine the roles of 3d TM dopants in 2D Ga_2_O_3_, focusing on structural stability, transition energies, magnetic properties, charge density variations, and electronic structures through first-principles calculations using the generalized gradient approximation (GGA) +U method. Different from the estimation of the defect formation energy solely in the neutral state, comprehensive evaluations considering different charge states are studied in the present calculations, which are crucial for determining the precise conductivity type (i.e., p-type or n-type). The shift in conductivity from n-type to p-type doping is suggested by the detailed variations in impurity levels within 3d TM-doped systems. Additionally, we investigate the contributions of 3d dopants to conductivity and spin features in 2D Ga_2_O_3_. The origin of the magnetic moment is further explained by employing partial density of states (PDOS) of 3d TM. Our results show that the 3d TM dopants can tune the conductive type from n-type to p-type and introduce ferromagnetism effectively in 2D Ga_2_O_3_ materials. This work may shed light on its feasible optoelectronic applications and offer theoretical guidance for the design of magnetic 2D Ga_2_O_3_ materials in potential DMS-based devices.

## 2. Computational Details

The Vienna ab initio Simulation Package (VASP) Standard Edition 6.4 has been utilized for first-principles calculations [29,30]. The exchange-correlation interactions are described by the projected augmented wave (PAW) potentials within the Perdew–Burke–Ernzerhof (PBE) functional, in the framework of a generalized gradient approximation (GGA) [31,32]. In this study, a 3 × 2 × 1 2D Ga_2_O_3_ supercell containing 60 atoms is modeled. The values of 450 eV, 1 × 10^−6^ eV/atom and 0.01 eV/Å are set for the criteria of energy cutoff, energy convergence, and residual forces, respectively. A resolution of 0.02 × 2π Å^−1^ is used for the k-point grid. Vacuum thickness of 15 Å is created along the c-axis direction to prevent spurious interactions between layers. The GGA+U approach is utilized for the 3d orbitals of TM dopants due to their strong Coulomb interactions, i.e., 3.3 [33], 2.3 [34], 3.1 [35], 3.5 [35], 4.0 [35], 4.0 [35], 3.3 [35], 6.4 [35], 7.0 [36], and 7.5 eV [37] for Sc, Ti, V, Cr, Mn, Fe, Co, Ni, Cu and Zn, respectively. These U values are U_eff_ values and U_eff_ = U-J, where U and J represent the Coulomb and exchange parameters, respectively. For comparison, electronic structure of undoped Ga_2_O_3_ is adjusted utilizing the screened Heyd–Scuseria–Ernzerh (HSE) hybrid density functional method with the mixing parameter of 35% to correct for the band gap [18].

**Figure 1:**
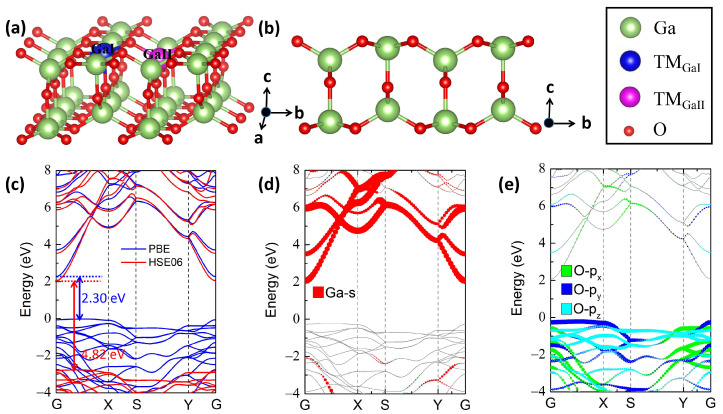
The structural model of 3d TM (Sc, Ti, V, Cr, Mn, Fe, Co, Ni, Cu, Zn)-doped 2D Ga_2_O_3_ structures from the (**a**) perspective view and (**b**) side view. The O and Ga atoms are represented by the smaller red and larger green spheres, respectively. The replaced GaI and GaII doping positions with a TM atom are indicated by the blue and magenta colors, respectively. (**c**) Band structure of perfect 2D Ga_2_O_3_ unit cell calculated by PBE and HSE06 functionals. Orbital-projected band structures of (**d**) Ga-4s orbitals and (**e**) O-2p orbitals for perfect 2D Ga_2_O_3_ unit cells under PBE functional. The high symmetry points are G (0, 0, 0), X (0.5, 0, 0), S (0.5, 0.5, 0), and Y (0, 0.5, 0), respectively.

Each bulk β-Ga_2_O_3_ contains octahedrally coordinated GaI and tetrahedrally coordinated GaII ions. β-Ga_2_O_3_ [100] direction possesses a larger lattice constant, which renders it easier to be cleaved, accompanied by low cleavage and formation energies of 1.11 J/cm^−1^ [38] and −5.76 eV [39], respectively. A stable 2D Ga_2_O_3_ monolayer is formed by cleaving the GaI–O bonds. Furthermore, GaI is restructured from six coordinated to five coordination, featuring a square pyramidal structure. Two inequivalent Ga sites, namely the square pyramidal GaI and the tetrahedral GaII, are thus presented in 2D Ga_2_O_3_. In this study, one 3d TM impurity, i.e., Sc, Ti, V, Cr, Mn, Fe, Co, Ni, Cu, Zn, substitutes for the GaI or GaII cation site and are labeled as TM_GaI_ and TM_GaII_, respectively, as illustrated in Figure 1a,b from perspective and side views, respectively.

When 2D Ga_2_O_3_ is doped with defect *D* in the charge state *q*, the defect formation energy is described as [40,41]

(1) $$H_{D,q}(E_{f},\mu )=[E_{D,q}-E_{p}]+\sum_{i}n_{i}\mu_{i}+q(E_{VBM}+E_{f})+E_{corr}$$

where $E_{D,q}$ and $E_{p}$ illustrate the energies in the doped and pure 2D Ga_2_O_3_ systems, respectively. $n_{i}$ demonstrates the quantity of *i* atom added ($n_{i}<0$) or extracted ($n_{i}>0$) from the pure 2D Ga_2_O_3_ system, and $\mu_{i}$ denotes the chemical potential. The chemical potential of $\mu_{\mathrm{TM}}$ and $\mu_{O}$ are gained from the stable bulk TM and O_2_, respectively. The related chemical potentials should satisfy the boundary conditions and categorize into the O-rich and Ga-rich cases in terms of the growth conditions, as stated in our previous studies [17,42]. $E_{VBM}$ shows the energy of *VBM* in the pure 2D Ga_2_O_3_. $E_{f}$ indicates the Fermi level, which is a variable between the *VBM* and conduction band minimum (*CBM*), that is, within a range of the band gap value. $E_{corr}$ is the corresponding finite-size effect, which can be obtained by the potential alignment [40]

(2) $$E_{corr}=q(V_{D,q}^{r}-V_{p}^{r})$$

Herein, ($V_{D,q}^{r}$) and ($V_{p}^{r}$) illustrate the atomic sphere-averaged electrostatic potential in the charged defect and pure 2D Ga_2_O_3_ structure, respectively, which are acquired using VASPKIT Standard Edition 1.3.5 software [43]. We note that the charge states of q used in the simulations vary for different TM-doped 2D Ga_2_O_3_. In this study, the +1 and neutral charge states are determined for the electron doping elements, i.e., Ti, V, Cr, Mn, Fe, Co, and Ni doping elements; the −1 and neutral charge states are suggested for the hole doping elements, i.e., Cu and Zn; and the +1 and neutral charge states are used for the Sc doping case.

The transition energy $\varepsilon (q_{1}/q_{2})$ between charge states $q_{1}$ and $q_{2}$ for defect *D* doping configuration is illustrated as [44]

(3) $$\varepsilon (q_{1}/q_{2})=\frac{E_{D}^{q_{1}}{|}_{E_{f}=0}-E_{D}^{q_{2}}{|}_{E_{f}=0}}{q_{2}-q_{1}}$$

where the $E_{D}^{q}\;{|}_{E_{f}=0}$ shows the formation energy of the defect *D* in charge state *q* determined at $E_{f}=0$.

## 3. Results and Discussion

### 3.1. Structural Stabilities

The lattice constants of pure 2D Ga_2_O_3_ derived from the bulk β-Ga_2_O_3_ material with the C2/m space group are a = 2.974 Å, b = 5.742 Å, which are in accordance with those values in Refs. [18,45,46]. The calculated optimized lattice parameters and atomic positions of the doped systems are shown in Table 1. The comparable lattice parameters between the 3d TM-doped systems and undoped systems demonstrate that the 3d-TM dopants produce relatively minor changes of atomic positions and crystal structures. The detailed percentage variations of the lattice constants for various dopants compared to perfect 2D Ga_2_O_3_ are shown in Figure S1a (Supplementary Materials). Overall, the relative variations in lattice constants a and b are less than 1%, regardless of the substitutions of TM ions at the square pyramidally coordinated GaI or the tetrahedrally coordinated GaII sites. Figure S1b in Supplementary Materials shows the variations, in sum, of the lattice constants a and b as the atomic number of 3d TM increases, where both doping sites exhibit decreasing tendencies. The variations can be ascribed to the changes of comparable local structures, as well as the ionic radii between the Ga and dopant. Moreover, as the atomic number of 3d TM increases, the differences of ionic radii between the foreign impurity and host Ga atom become smaller, which agrees well with the resolved variations of the lattice constants.

Figure 1c indicates the band structures of pure 2D Ga_2_O_3_ calculated by PBE and HSE06 functionals. The band gaps of perfect 2D Ga_2_O_3_ calculated by PBE and HSE functionals are 2.30 and 4.82 eV, which are consistent with previous values of 2.28 eV [45] and 4.69 eV [46], respectively. As shown in Figure 1c, the CBM and VBM of pure 2D Ga_2_O_3_ are respectively located at the G point and non-G point (between the G and X points) for both PBE and HSE calculations, demonstrating an indirect band-gap semiconductor nature. Note that the common occurrence of an underestimated band gap using PBE calculations does not impact our conclusions qualitatively [47,48]. Additionally, the energy difference between the indirect and the direct band gaps is minimal (approximately 0.04 eV) in both situations. The CBM and VBM consist mainly of Ga-4s orbitals and O-2p orbitals, as shown in Figure 1d and e, respectively. It is difficult to achieve p-type conductivity due to flat VBM, whereas n-type doping is simpler in terms of the delocalized dispersion features of CBM in 2D Ga_2_O_3_.

**Figure 2:**
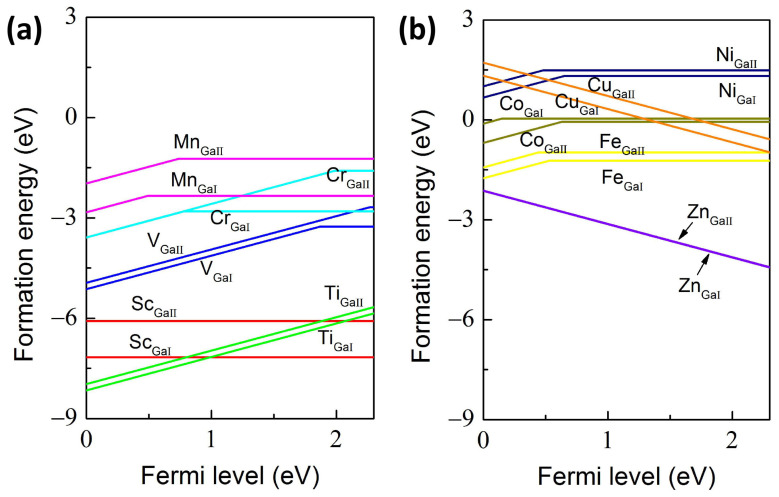
The calculated (**a**,**b**) defect formation energies for different TM-doped 2D Ga_2_O_3_ structures under O-rich conditions.

The structural stability for different TM-doped 2D Ga_2_O_3_ are evaluated using the defect formation energies and transition levels, as depicted in Figure 2. The Fermi level is variable within the band gap values from 0 to 2.30 eV, i.e., from VBM to CBM. Firstly, these TM-doped configurations possess a lower formation energy under O-rich conditions than under Ga-rich conditions, suggesting that these TM dopants replacing Ga sites more easily form in the O-rich environment, which is consistent with other results in TM-doped 3D Ga_2_O_3_ [22,23]. This can be attributed to the challenge of forming Ga vacancy in Ga-rich conditions. Therefore, this study discusses TM-doped cases only under O-rich conditions. Secondly, as shown in Figure 2, the Co_GaII_ case has a lower formation energy compared to that of Co_GaI_ in the entire band gap region, indicating that the occupation of Co impurity at the GaII site is energetically favored over the GaI doping site; whereas for other dopants, the substitutions of the GaI site exhibit lower formation energies compared to that of the GaII site, demonstrating that these dopants tend to occupy the GaI sites. Therefore, the Co impurity preferentially incorporates on the tetrahedral GaII site, while the other dopants favor square pyramidal GaI sites in 2D Ga_2_O_3_. Thus, we mainly discuss these stable systems afterwards, i.e., Sc_GaI_, Ti_GaI_, V_GaI_, Cr_GaI_, Mn_GaI_, Fe_GaI_, Co_GaII_, Ni_GaI_, Cu_GaI_, and Zn_GaI_. Thirdly, in view of the different electronic configurations between host Ga and TM atoms, i.e., three electrons in the outermost layers for host Ga atom, three electrons for Sc impurity, more than three outermost electrons for Ti, V, Cr, Mn, Fe, Co, Ni, as well as fewer than three electrons for Cu and Zn in the outmost shells, the TM_Ga_ configurations can be grouped into three categories, i.e., the neutral doped Sc_GaI_ case, the electron doping cases including Ti_GaI_, V_GaI_, Cr_GaI_, Mn_GaI_, Fe_GaI_, Co_GaII_, and Ni_GaI_, as well as the hole doping cases containing Cu_GaI_ and Zn_GaI_. The formation energy of Sc_GaI_ is calculated as −7.17 eV, experimentally suggesting that the Sc element can spontaneously incorporate on the GaI atom in 2D Ga_2_O_3_. For these electron doping cases, Ti_GaI_ is easily formed, followed by V_GaI_, Cr_GaI_, Mn_GaI_, Fe_GaI_, Co_GaII_, and Ni_GaI_. Therefore, as the number of electrons in the outmost shells increases, the formation energies increase. Moreover, these electron doping structures, with the exception of Ni_GaI_, are characterized by the negative formation energies in the entire band gap region, experimentally suggesting that these 3d TM elements except for Ni can easily substitute for the host Ga atom in 2D Ga_2_O_3_. For the hole doping cases, one can notice that the Zn_GaI_ is characterized by a lower formation energy than that of Cu_GaI_, demonstrating that the foreign Zn atom more readily replaces the Ga atom compared to the Cu atom.

Finally, the detailed transition levels are calculated, which are important in evaluating the charge states and the deep/shallow donor and acceptor ionization energy levels. For the Sc_GaI_ configuration, the 0 charge state, i.e., the Sc^3+^ oxidation state, is anticipated in the entire band gap region, as indicated in Figure 2a. For the Ti_GaI_ combination, the +1 charge state is energetically favored over the whole band gap, suggesting that the n-type conductivity can be enhanced by the replacement of the GaI atom by the Ti impurity. The transition levels ε(+1/0) are located at 1.87, 0.78, 0.49, 0.53, 0.63, and 0.65 eV above the VBM for the V_GaI_, Cr_GaI_, Mn_GaI_, Fe_GaI_, Co_GaII_, and Ni_GaI_ systems, respectively, indicating that these dopants behave with n-type conductivity and undergo the transition from the relative shallow donor to deep donor doping levels. Furthermore, as the Fermi levels get close to the VBM and CBM, the +1 and 0 charge states are expected, respectively. For the Cu_GaI_ and Zn_GaI_ structures, as shown in Figure 2b, the charge state of −1 is energetically favorable throughout the entire band gap range, which illustrates that the Cu_GaI_ and Zn_GaI_ structures act at shallow acceptor levels and are dominated by Cu^2+^ and Zn^2+^ oxidation states, respectively. The shallow impurity levels in Cu- and Zn-doped 2D Ga_2_O_3_ are compared and consistent with the values in their 3D counterparts [19,21], as shown in Figure 2b, the energy difference between Zn_GaI_ and Zn_GaII_ is only 0.04 eV, indicating that Zn_GaII_ is an alternative optional doping structure. Therefore, our results suggest that a transition from n-type to p-type conductivity occurs at the threshold Cu element, which can be ascribed to the shift from electron doping to hole doping with respect to the increase in the atomic number in the 3d TM group. It is worth noting that some intrinsic defects, such as V_O_ and Ga interstitial (Ga_i_), are unintentionally created during the growth of Ga_2_O_3_, generating n-type conduction nature [11]. Therefore, the Fermi level is typically located at the upper region of the band gap in Ga_2_O_3_. As a result, our calculated results predict that the predominant charge states in 2D Ga_2_O_3_ upon TM doping are 0, +1, +1, 0, 0, 0, 0, 0, −1, and −1 for Sc^3+^, Ti^4+^, V^4+^, Cr^3+^, Mn^3+^, Fe^3+^, Co^3+^, Ni^3+^, Cu^2+^ and Zn^2+^, respectively.

### 3.2. Magnetic and Electronic Properties

**Figure 3:**
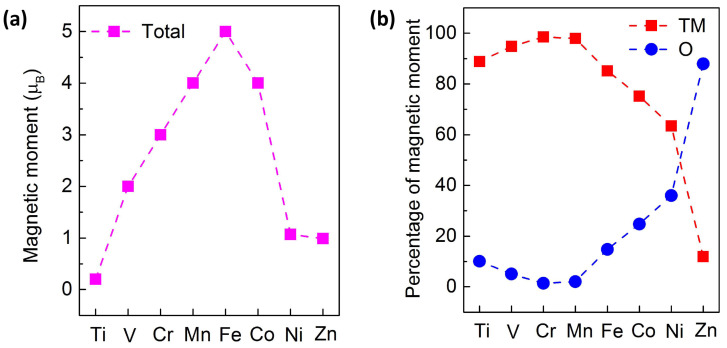
(**a**) The magnetic moments for TM-doped 2D Ga_2_O_3_ structures and (**b**) their corresponding percentage contributions of TM and O ions to the total magnetic moments versus different TM dopants.

TM dopants can induce and tune local magnetism. As indicated in Figure 3a, among these TM doping configurations, the Sc- and Cu-doped 2D Ga_2_O_3_ systems exhibit nonmagnetic ground states, while the other cases exhibit ferromagnetic states. The total magnetic moments for Ti-, V-, Cr-, Mn-, Fe-, Co-, Ni-, and Zn-doped 2D Ga_2_O_3_ structures are calculated as 0.2, 2.0, 3.0, 4.0, 5.0, 4.0, 1.1, and 1.0 μ_B_, respectively.

**Figure 4:**
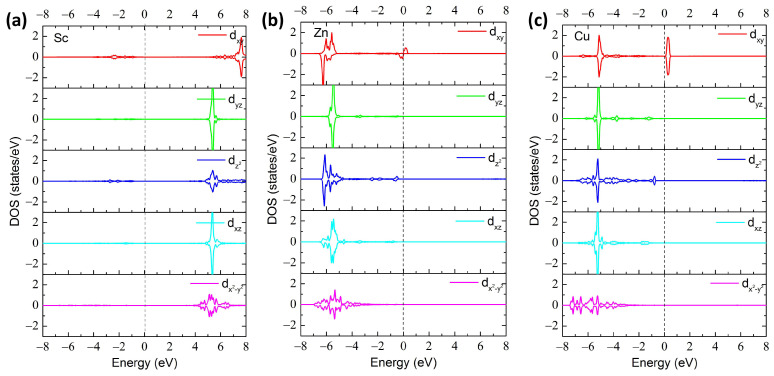
Calculated PDOS of TM-3d orbitals for (**a**) Sc_GaI_, (**b**) Zn_GaI_, and (**c**) Cu_GaI_ structures.

In order to explore the origin of the induced magnetic moment, the partial density of states (PDOS) are employed. In common with the host Ga atom, the Sc atom possesses three available electrons that are used to form bonds with neighboring O atoms when replacing the Ga atom; thus, no extra free electron remains, resulting in a nonmagnetic ground state. Figure 4a shows the PDOS of impurity Sc in 2D Sc_GaI_ Ga_2_O_3_. The distributions of the unoccupied states in the spin-up and spin-down channels are completely symmetrical, suggesting a nonmagnetic nature. For the hole doping cases, the Zn atom possesses two free electrons. One additional hole doping is thus anticipated by the replacing of a Ga atom by a Zn foreigner, which agrees well with the magnetic moment of 1.0 μ_B_ for the Zn-doped systems [26]. The extra hole can be further associated with the spin-up orbital of d_xy_ symmetry situated in the conduction band, as shown in Figure 4b, giving rise to a magnetic moment of 1.0 μ_B_ for the Zn_GaI_ configuration. Similarly, Cu dopant generates two extra holes located in the spin-up and spin-down channels of Cu-d_xy_ orbitals, which are symmetrically distributed near the Fermi level, and thus no additional magnetic moment is created, as is shown in Figure 4c. The low-spin electronic configuration with a nonmagnetic nature differs from the high-spin electronic configuration with a magnetic moment of 2.0 μ_B_ in Cu-doped bulk β-Ga_2_O_3_ [19], which can be attributed to the various local crystal structures, i.e., the square pyramidal coordinated GaI in 2D Ga_2_O_3_ and octahedral coordinated GaI in 3D Ga_2_O_3_.

**Figure 5:**
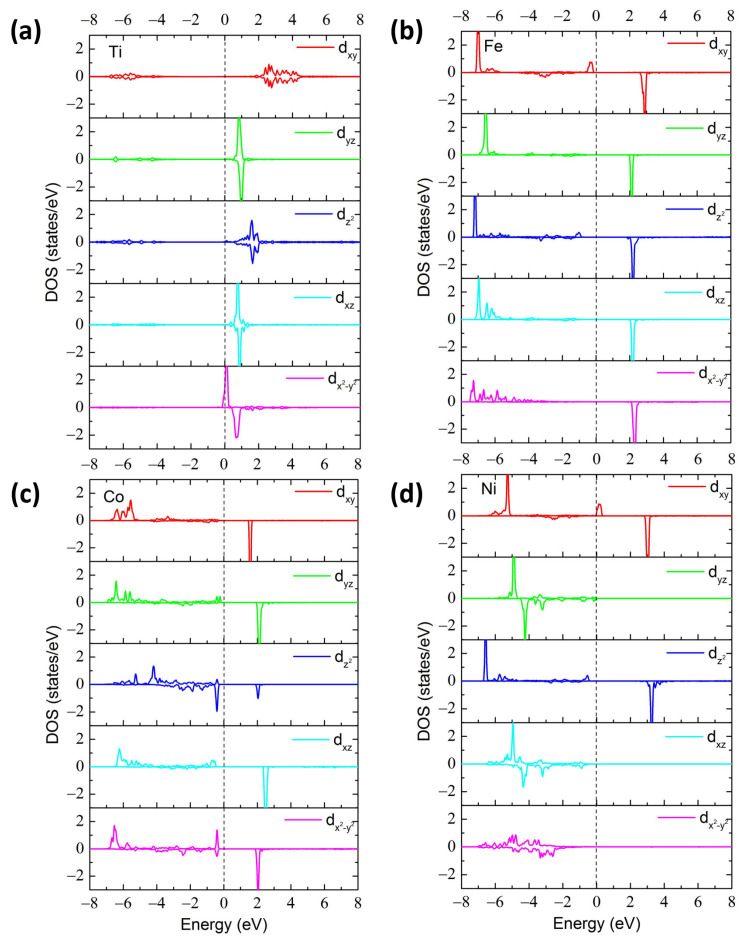
Calculated PDOS of TM-3d orbitals for (**a**) Ti_GaI_, (**b**) Fe_GaI_, (**c**) Co_GaII_, and (**d**) Ni_GaI_ structures.

For the electron doping cases, the magnetic moment of the Ti_GaI_ structure is 0.2 μ_B_, which is a comparable value with 0.546 μ_B_ in Ti-doped bulk β-Ga_2_O_3_ [28]. The reason is that the Ti atom possesses four free electrons, three of which are formed bonds with neighboring O atoms accompanying the 3d^1^ valence electron of the Ti^3+^ ion, whereas the remaining unpaired electron is expected to produce a magnetic moment of 1.00 μ_B_. However, the Ti ion tends to give the extra electron to form the +4 valence state as suggested by the formation energy analysis. Therefore, as depicted in Figure 5a, the spin-down orbitals of foreign Ti are all unoccupied, while the spin-up state of the Ti-d_x_^2^_-y_^2^ orbital is only partially occupied, resulting in the small magnetic moment of 0.2 μ_B_. Our calculated magnetic moments for V_GaI_, Cr_GaI_, and Mn_GaI_ cases are 2.0, 3.0, and 4.0 μ_B_, respectively, which are in accordance with the values in the Ref. [25] and can be ascribed to the fully occupied orbitals of V-d_x_^2^_-y_^2^ and d_xz_, Cr-d_x_^2^_-y_^2^, d_xz_, and d_yz_, and Mn-d_x_^2^_-y_^2^, d_xz_, d_yz_, and d_z_^2^, respectively (as shown in Figure S2 of Supplementary Information).

In the case of the Fe dopant, the Fe atom possesses eight free electrons, three of which are provided to form bonds with neighboring O atoms accompanying the 3d^5^ valence electron of Fe^3+^ ion, whereas the five unpaired electrons lead to total magnetic moments of 5.0 μ_B_, suggesting a high-spin electronic configuration. The PDOS of the 3d-orbitals of the Fe_GaI_ structure is depicted in Figure 5b. The five spin-up and spin-down states are situated in the valence and conduction bands, respectively, giving rise to a total magnetic moment of 5.0 μ_B_. For the Co_GaII_ case with nine electrons, three of which are provided to form bonds with neighboring O atoms and the remaining six electrons. The PDOS of the 3d-orbitals of Co_GaII_ structure is depicted in Figure 5c, which is demonstrated as a high-spin electronic configuration with the spin-up channels fully occupied and one extra electron occupying the Co-d_z_^2^ orbital. Thus, the four unpaired electrons in the Co-d_x_^2^_-y_^2^, d_xz_, d_yz_, and d_xy_ orbitals generate a magnetic moment of 4.0 μ_B_ in the Co_GaII_ system. For the Ni-doped 2D Ga_2_O_3_ combination, seven free electrons are expected. Figure 5d illustrates that six free electrons located at Ni-d_x_^2^_-y_^2^, d_xz_, and d_yz_ are paired and one electron occupying the orbital of Ni-d_z_^2^ is unpaired, corresponding to a low-spin electronic configuration therein. Therefore, a total magnetic moment of about 1.0 μ_B_ is produced when one Ni impurity substitutes the host GaI site. Therefore, in terms of magnetic moments, V, Cr, Mn, and Zn dopants exhibit similar contributions in 3D and 2D Ga_2_O_3_, whereas the magnetic properties of Cu-doped 3D and 2D Ga_2_O_3_ structures are completely different [25,26]. Cu-doped 3D Ga_2_O_3_ possesses a magnetic moment of 2 μ_B_ with a high-spin state, whereas Cu-doped 2D Ga_2_O_3_ is characterized here by a nonmagnetic property in a low-spin configuration [19]. Thus, different structures give rise to different findings.

**Figure 6:**
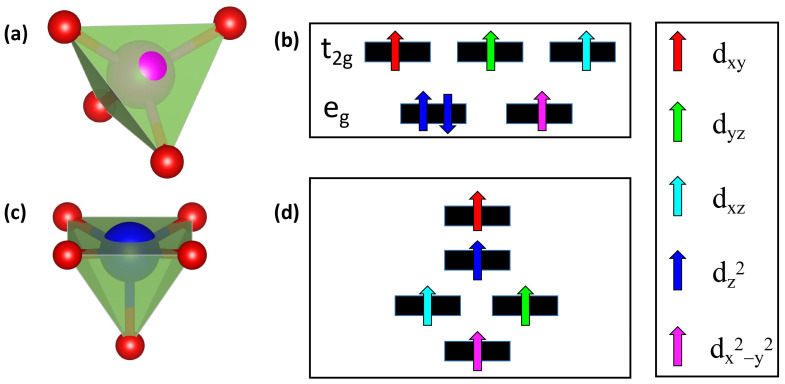
Illustrations of the (**a**) tetrahedral GaII and the (**c**) square pyramidal GaI coordinated crystal structures. Panels (**b**) and (**d**) show the spin arrangements for Co_GaII_ and Fe_GaI_ systems, respectively.

The spin configurations for different TM-doped structures can be fully understood from crystal field effects. As mentioned above, the Co dopants tend to reside on the tetrahedral GaII site, whereas the other 3d TM dopants typically occupy the square pyramidal GaI sites. For the Co_GaII_ structure, as illustrated in Figure 6a, the tetrahedral crystal field leads to the low-lying e_g_ orbital levels and the high-lying t_2g_ orbital levels. The six extra electrons follow the high-spin arrangements, resulting in a total magnetic moment of 4.0 μ_B_, as shown in Figure 6b. For the square pyramidal coordinated structures composed of one TM atom and five O atoms, as illustrated in Figure 6c, the missing Ga-O bond along [100] direction breaks the octahedral crystal field. Our results indicate an orbital rearrangement, as shown in Figure 6d, where the d_x_^2^_-y_^2^, d_xz_ (d_yz_), d_z_^2^, and d_xy_ orbitals are arranged from low to high energy levels. The broken octahedral crystalline field leads to the splitting of d orbitals, as observed in other systems such as TM-doped SnTe [49]. Taking the Fe_GaI_ as an example, the five unpaired electrons occupy each orbital level with a high-spin state, giving rise to a magnetic moment 5.00 μ_B_, as shown in Figure 6d. The detailed spin arrangements for various TM-doped 2D Ga_2_O_3_ structures are summarized in Table 2. Our results show that the dopants from V to Co are high-spin states, while the Ni and Cu dopants possess low-spin configurations. In other words, a transition from high-spin to low-spin arrangement is observed.

**Figure 7:**
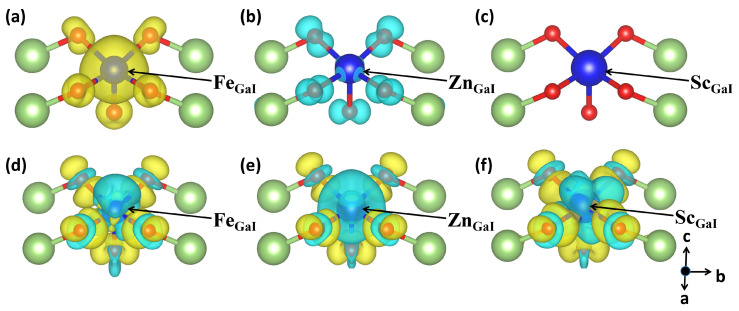
The spin densities for (**a**) Fe_GaI_, (**b**) Zn_GaI_, and (**c**) Sc_GaI_ structures in 2D Ga_2_O_3_. The spin-up density is designated as yellow, and the spin-down density is labeled as cyan. The charge density differences for (**d**) Fe_GaI_, (**e**) Zn_GaI_, and (**f**) Sc_GaI_ structures in 2D Ga_2_O_3_. Charge accumulation and depletion are indicated by the yellow and cyan regions, respectively. The value of isosurface level is set as 0.005 e/Å^3^ for spin densities and charge density differences.

To provide insights into the quantitative contributions of the total magnetic moment from TM and O ions, the percentage contribution of individual terms has been estimated, as depicted in Figure 3b. One can notice that the TM atoms are the primary source of the magnetic moment qualitatively, while the host O exhibits comparatively tiny localized magnetic moments when the atomic number of the TM dopants is relatively small. However, the contribution of O atoms to the induced magnetic moment increases remarkably as the atomic number of the 3d TM dopant increases, which can be attributed to the increased electronegativity from Sc to Zn atoms. Moreover, the contribution from the Ga atoms is relatively small. Therefore, as the atomic number of the 3d TM dopant increases, the percentage contribution of the total magnetic moment induced by the TM atoms greatly decreases, while the percentage provided by the O atoms significantly increases. The detailed distributions of the spin densities for different doping combinations are also considered here; among them three representative cases are presented in Figure 7a–c, i.e., the electron doping configuration of Fe-doped 2D Ga_2_O_3_ with the highest magnetic moment, the hole doping configuration of Zn-doped 2D Ga_2_O_3_ with the highest magnetism, and the Sc-doped 2D Ga_2_O_3_, respectively. The spin-up density is designated as yellow, and the spin-down density is labeled as cyan. For the Fe_GaI_ case, we can clearly observe from Figure 7a that the foreign Fe atom is the qualitative source of the majority of the magnetic moment. The contribution from the neighboring O atoms is relatively small, and the effect of the Ga atoms on the magnetic moment is negligible. However, as depicted in Figure 7b for the Zn_GaI_ structure, the majority magnetic moments are qualitatively originated from the most adjacent five O atoms, rather than the Zn impurity. Figure 7c indicates the absence of a magnetic moment in the case of Sc_GaI_.

Furthermore, the calculations of charge density differences and Bader charges are also employed for these three representative cases to evaluate the charge transformations and redistributions, as shown in Figure 7d–f. Charge accumulation and depletion are indicated by the yellow and cyan regions, respectively. As shown in Figure 7d–f, significant charge loss around the TM dopants and the modest charge accumulations from the adjacent O atoms are anticipated for the cases of Fe_GaI_, Zn_GaI_, and Sc_GaI_, indicating the electrostatic interactions between the O atoms and the TM element. The Bader charge analyses denote that the extrinsic Fe, Zn, and Sc dopants lose 1.75e, 1.30e, and 2.14e charge to the systems, respectively. Therefore, positive charge states for these TM ions are expected, which agrees with the results obtained from formation energies. The Bader charges for other doping structures are also summarized in Table S1 (Supplementary Information), where all the doping systems lose electrons ranging from 1.30e to 2.47e charge.

**Figure 8:**
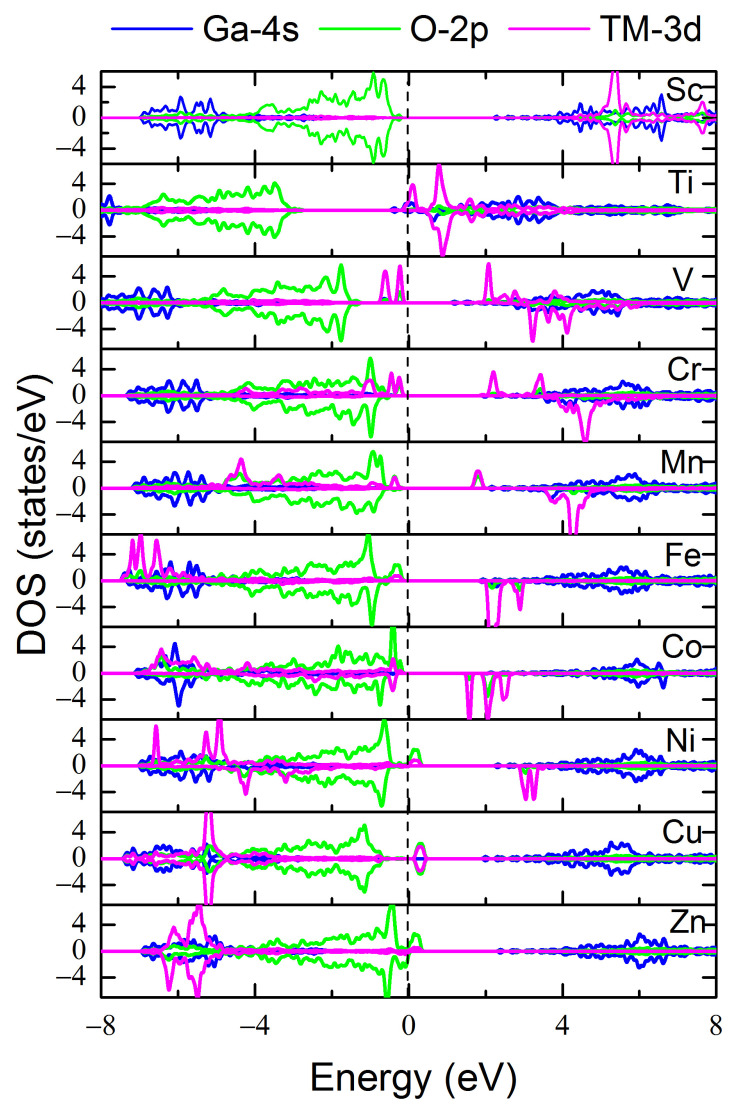
PDOS of the O 2p, TM 3d, and Ga 4s orbitals for the TM-doped 2D Ga_2_O_3_ systems. The Fermi level is labeled by a dashed line. The O 2p and Ga 4s orbitals are amplified five and ten times, respectively, for clarity.

The PDOS of the O 2p, TM 3d, and Ga 4s orbitals for all the defect cases in the 2D Ga_2_O_3_ systems are shown in Figure 8. As the electronegativity of the doped 3d TM element increases, the Fermi level changes from CBM to VBM, demonstrating a transformation from n-type to p-type conductivity, which verifies the results obtained from formation energy analyses. This can be ascribed to the change from introducing electron/electrons to introducing hole/holes. The 3d orbitals of Sc and Ti impurities hybridize with the Ga-4s orbitals at the CBM. As the dopant atomic number increases, the 3d orbitals of the TM tend to hybridize with the O 2p orbitals at the VBM, which can be ascribed to the lower atomic 3d orbital energy levels as the 3d TM atomic number increases. Therefore, the overlaps between the 3d states of the TM and those of the O-2p are enhanced with the increase of the dopant atomic number. Moreover, the formed 3d bands for most TM dopants are located near the Fermi level, which can significantly benefit the transformation of the absorbing region from ultraviolet to visible/infrared light. The detailed band gaps and band structures of the TM-doped structures including the spin-up and spin-down are summarized in Table S2 and Figure S3, respectively, in Supplementary Materials.

Moreover, in view of the magnetism and the band structures obtained from current calculations, we can classify these dopants into three categories, i.e., nonmagnetic semiconductors, including Sc and Cu; ferromagnetic semiconductors, including V, Cr, Mn, Fe, Co, Ni, and Zn; and ferromagnetic half-metals, including Ti. These functional properties may provide design guidance for various spintronic and optoelectronic applications based on 2D Ga_2_O_3_ material.

### 3.3. Conclusions

In this study, the generalized gradient approximation (GGA) +U method, based on first-principles calculations, was used to investigate the role of 3d TM-doped 2D Ga_2_O_3_, including its structural stability, transition energy levels, magnetic properties, charge densities, and electronic structures. Our results show that Co impurities energetically tend to replace GaII sites, while the other 3d TM impurities tend to occupy GaI sites. Defect formation energies indicate from experiments that Sc can spontaneously incorporate on the GaI atom in 2D Ga_2_O_3_. For electron doping, Ti_GaI_ is the most easily formed, followed by V_GaI_, Cr_GaI_, Mn_GaI_, Fe_GaI_, Co_GaII_, and Ni_GaI_. In the hole doping structures, the Zn atom more easily replaces the Ga atom compared to the Cu atom. Our calculations predict that Sc^3+^, Ti^4+^, V^4+^, Cr^3+^, Mn^3+^, Fe^3+^, Co^3+^, Ni^3+^, Cu^2+^, and Zn^2+^, are the predominant states in 2D Ga_2_O_3_. Importantly, a transition from n-type to p-type conductivity occurs as the atomic number of 3d TM dopant increases, based on defect formation energies and the PDOS, which can be ascribed to the shift from electron doping to hole doping with respect to the increase in the atomic number of 3d TM. The Sc- and Cu-doped 2D Ga_2_O_3_ systems exhibit nonmagnetic ground states, while the other cases exhibit ferromagnetic states. The total magnetic moments for Ti-, V-, Cr-, Mn-, Fe-, Co-, Ni-, and Zn-doped 2D Ga_2_O_3_ structures are calculated as 0.2, 2.0, 3.0, 4.0, 5.0, 4.0, 1.1, and 1.0 μ_B_, respectively. Moreover, the spin configurations in the presence of the square pyramidal and tetrahedral coordinated crystal field effects are investigated in detail, and a transition from high-spin to low-spin arrangement is observed. As the atomic number of the 3d TM dopant increases, the percentage of magnetic moments induced by the TM atom greatly decreases, while the percentage provided by the O atom significantly increases. As the electronegativity of the dopant rises, the 3d states of the TM overlap significantly with those of the O-2p states. Additionally, the formed 3d bands for most TM dopants are located near the Fermi level, which can significantly benefit the transformation of the absorbing region from ultraviolet to visible/infrared light.

## Figures and Tables

**Table 1:** The calculated lattice constants and atomic position of TM for perfect and TM-doped 2D Ga_2_O_3_.

Structure	Lattice Constants		Atomic Position of TM_GaI_	Structure	Lattice Constants		Atomic Position of TM_GaII_
	a (Å)	b (Å)			a (Å)	b (Å)	
Perfect	2.974	5.742	(0.667, 0.499, 0.204)	Perfect	2.974	5.742	(0.500, 0.749, 0.173)
Sc_GaI_	3.003	5.768	(0.667, 0.499, 0.209)	Sc_GaII_	2.988	5.779	(0.500, 0.749, 0.181)
Ti_GaI_	2.997	5.784	(0.667, 0.499, 0.200)	Ti_GaII_	2.996	5.781	(0.500, 0.749, 0.173)
V_GaI_	2.992	5.755	(0.667, 0.499, 0.206)	V_GaII_	2.983	5.763	(0.500, 0.749, 0.181)
Cr_GaI_	2.989	5.749	(0.667, 0.499, 0.207)	Cr_GaII_	2.984	5.770	(0.500, 0.749, 0.181)
Mn_GaI_	2.985	5.745	(0.667, 0.499, 0.213)	Mn_GaII_	2.988	5.779	(0.500, 0.750, 0.183)
Fe_GaI_	2.987	5.752	(0.667, 0.499, 0.205)	Fe_GaII_	2.983	5.756	(0.500, 0.749, 0.175)
Co_GaI_	2.986	5.750	(0.667, 0.499, 0.201)	Co_GaII_	2.982	5.755	(0.500, 0.749, 0.178)
Ni_GaI_	2.986	5.743	(0.667, 0.499, 0.204)	Ni_GaII_	2.981	5.742	(0.500, 0.749, 0.176)
Cu_GaI_	2.974	5.730	(0.667, 0.499, 0.217)	Cu_GaII_	2.987	5.749	(0.500, 0.749, 0.182)
Zn_GaI_	2.984	5.738	(0.667, 0.499, 0.209)	Zn_GaII_	2.984	5.734	(0.500, 0.749, 0.176)

**Table 2:** The spin occupations for different TM-doped 2D Ga_2_O_3_ structures.

Structure	d_x_^2^−_y_^2^	d_xz_	d_yz_	d_z_^2^	d_xy_
Sc_GaI_	-	-	-	-	-
Ti_GaI_	↑	-	-	-	-
V_GaI_	↑	↑	-	-	-
Cr_GaI_	↑	↑	↑	-	-
Mn_GaI_	↑	↑	↑	↑	-
Fe_GaI_	↑	↑	↑	↑	↑
Co_GaI_	↑	↑	↑	↑↓	↑
Ni_GaI_	↑↓	↑↓	↑↓	↑	-
Cu_GaI_	↑↓	↑↓	↑↓	↑↓	-
Zn_GaI_	↑↓	↑↓	↑↓	↑↓	↑

## Data Availability

Data are contained within the article and Supplementary Materials.

## Supplementary Materials

The following supporting information can be downloaded at: https://www.mdpi.com/article/10.3390/ma17184582/s1, Figure S1: The relative variations in lattice constants (a) a and b, and (b) a+b for 3d TM-doped 2D Ga2O3 structures, respectively; Figure S2: Calculated PDOS contributed by TM-3d orbitals for (a) V_GaI_, (b) Cr_GaI_, and (c) Mn_GaI_ structures; Table S1: The calculated Bader charge transfer of TM for different TM-doped 2D Ga_2_O_3_ configurations. Positive and negative values of Bader charge in unit of e denote acquire and loss electrons, respectively; Table S2: The calculated band gaps for perfect and TM-doped 2D Ga_2_O_3_ in unit of eV; Figure S3: The band structures of different TM-doped 2D Ga_2_O_3_ structures.
