# Supplementary material for: Roles of Impurity Levels in 3d Transition Metal-Doped Two-Dimensional Ga2O3
Source: Materials (Basel). 2024 Sep 18;17(18):4582. doi: 10.3390/ma17184582 (PMC11433155; doi:10.3390/ma17184582)
Supplement: Supplementary file 1 [file materials-17-04582-s001.zip › materials-3182171-supplementary.pdf]

materials

Supplementary Materials

Roles of Impurity Levels in 3d Transition Metal-Doped Two-Dimensional Ga_2O_3

Hui Zeng [1,2,*], Chao Ma [2], Xiaowu Li [1], Xi Fu [1], Haixia Gao [1] and Meng Wu [3,*]

1 College of Science, Hunan University of Science and Engineering, Yongzhou 425199, China; lixiaowu555@163.com (X.L.); fxhuse@huse.edu.cn (X.F.); haixiagao@huse.edu.cn (H.G.)
2 College of Materials Science and Engineering, Hunan University, Changsha 410082, China; cma@hnu.edu.cn
3 Fujian Provincial Key Laboratory of Semiconductors and Applications, Collaborative Innovation Center for Optoelectronic Semiconductors and Efficient Devices, Department of Physics, Xiamen University, Xiamen 361005, China
* Correspondence: 19820170155498@stu.xmu.edu.cn (H.Z.); meng.wu@xmu.edu.cn (M.W.)

Citation: Zeng, H.; Ma, C.; Li, X.; Fu, X.; Gao, H.; Wu, M. Roles of Impurity Levels in 3d Transition Metal-Doped Two-Dimensional Ga_2O_3. *Materials* **2024**, *17*, x. https://doi.org/10.3390/xxxxx

Academic Editors: Nikolas J. Podraza and Adolfo Avella

Received: 14 August 2024
Revised: 12 September 2024
Accepted: 16 September 2024
Published: date

Copyright: © 2024 by the authors. Submitted for possible open access publication under the terms and conditions of the Creative Commons Attribution (CC BY) license (https://creativecommons.org/licenses/by/4.0/).

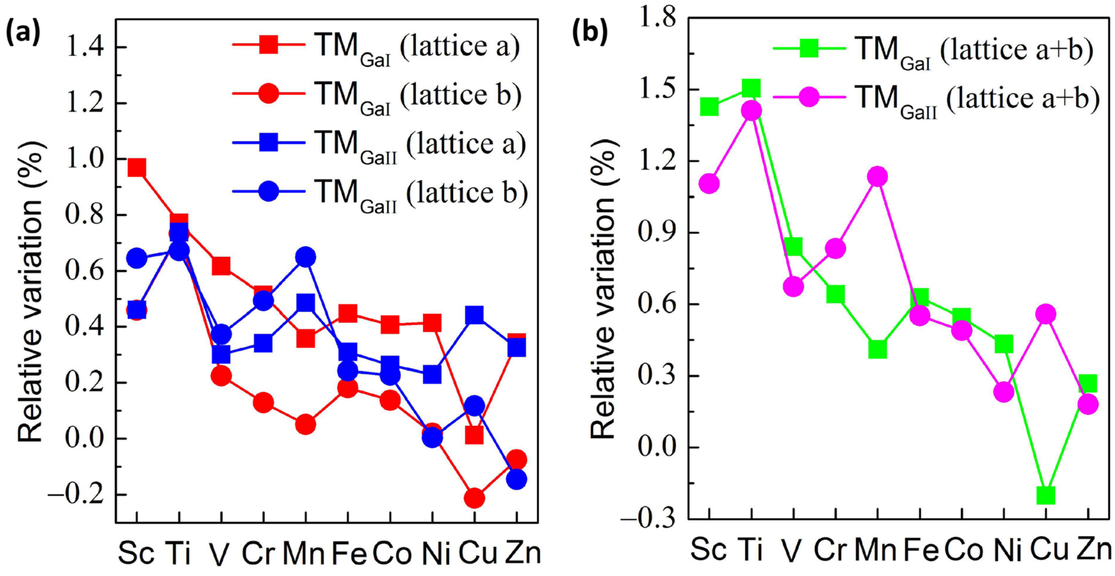

Figure S1. The relative variations in lattice constants (**a**) a and b, and (**b**) a+b for 3d TM-doped 2D Ga_2O_3 structures, respectively.

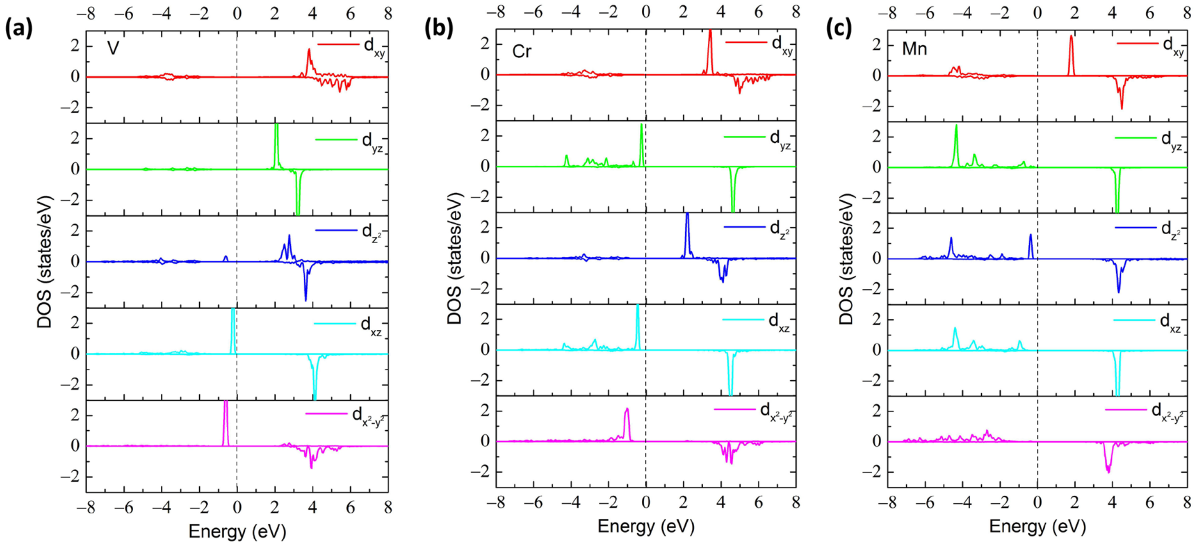

Figure S2. Calculated PDOS contributed by TM-3d orbitals for (**a**) V_{GaI}, (**b**) Cr_{GaI}, and (**c**) Mn_{GaI} structures.

Table S1. The calculated Bader charge transfer of TM for different TM-doped 2D Ga_2O_3 configurations. Positive and negative values of Bader charge in unit of *e* denote acquire and loss electrons, respectively.

Configurations	Bader Charge (e)
Sc_{GaI}	−2.14
Ti_{GaI}	−2.24
V_{GaI}	−1.92
Cr_{GaI}	−1.30
Mn_{GaI}	−1.75
Fe_{GaI}	−1.75
Co_{GaII}	−1.47
Ni_{GaI}	−2.47

Cu_{GaI}	−1.18
Zn_{GaI}	−1.30

Table S2. The calculated band gaps for perfect and TM-doped 2D Ga_2O_3 in unit of eV.

	Band Gap (eV)	
**Configurations**	**Spin-Up**	**Spin-Down**
Perfect	2.30	2.30
Sc_{GaI}	2.38	2.38
Ti_{GaI}	2.31	2.32
V_{GaI}	1.30	2.40
Cr_{GaI}	2.05	2.40
Mn_{GaI}	1.89	2.27
Fe_{GaI}	2.03	2.36
Co_{GaII}	2.23	1.90
Ni_{GaI}	0.01	2.22
Cu_{GaI}	0.35	0.35
Zn_{GaI}	0.04	2.29

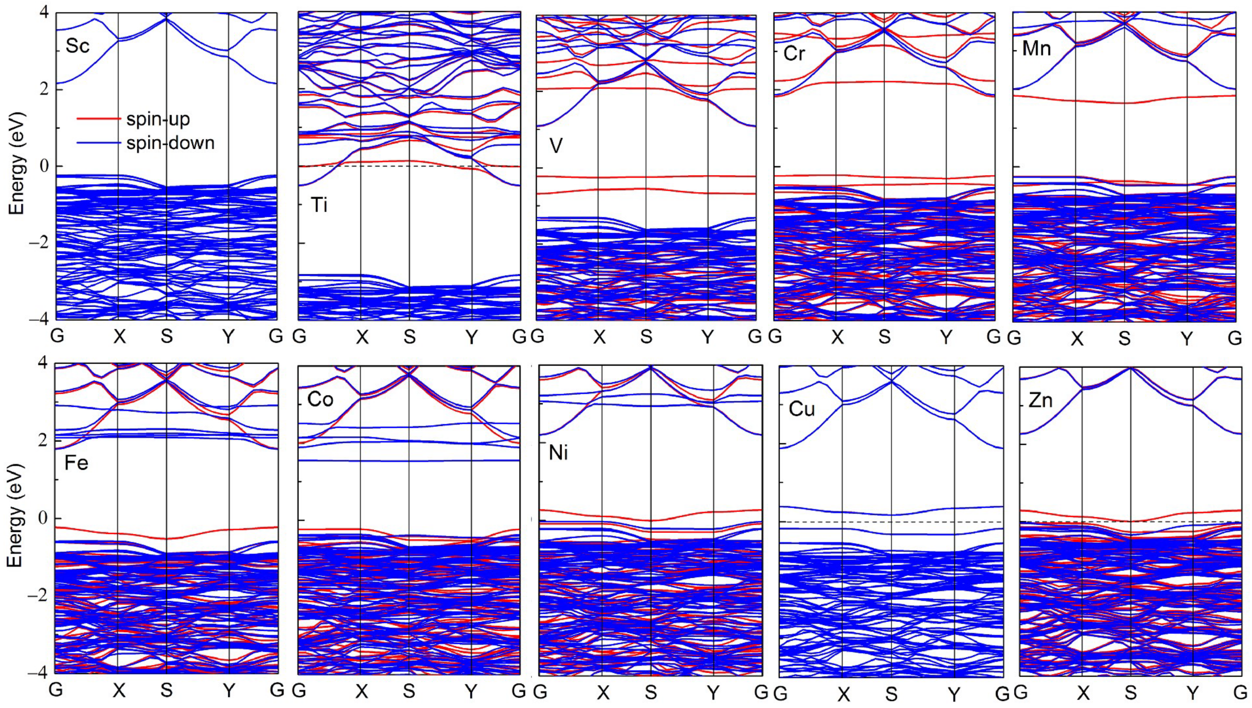

Figure S3. The band structures of different TM-doped 2D Ga_2O_3 structures.
